# Impact of COVID-19 pandemic on a daily-based outpatient treatment routine: experience of a radiotherapy department of a tertiary public/university hospital in Brazil

**DOI:** 10.6061/clinics/2020/e2298

**Published:** 2020-11-02

**Authors:** Heloisa de Andrade Carvalho, Karina Gondim Moutinho C. Vasconcelos, Herbeni Cardoso Gomes, João Víctor Salvajoli

**Affiliations:** Instituto do Cancer do Estado de Sao Paulo (ICESP), Instituto de Radiologia (InRad), Serviço de Radioterapia, Hospital das Clinicas (HCFMUSP), Faculdade de Medicina, Universidade de Sao Paulo, SP, BR

**Keywords:** Radiotherapy, Coronavirus Pandemic, Treatment Policy

## Abstract

**OBJECTIVES:**

To report the impact of the COVID-19 pandemic on patient attendance at a radiotherapy department two months after the implementation of specific policies regarding the pandemic.

**METHODS:**

The proposed treatment schemes, favoring hypofractionated schedules, and COVID-19 management strategies regarding irradiation are presented. Attendance after two months of implementation of these policies was measured and compared with that during the same period in 2019.

**RESULTS:**

A 10% reduction in the number of treated patients and a 26% reduction in the number of sessions was observed. The main impact was a decrease in the treatment of benign diseases and gastrointestinal tumors, with a general increase in breast cancer treatments. Eighteen (1.7%) patients were confirmed as having COVID-19 during radiotherapy in April and May 2020, three of whom were hospitalized, and one patient died because of COVID-19. Among the 18 patients, 12 had their treatments interrupted for at least 15 days from symptom appearance.

**CONCLUSION:**

There was a decrease in the number of treated patients in our radiotherapy department, with a greater decrease in the total number of sessions. This indicated, overall, a smaller number of fractions/patients treated, despite our efforts to maintain the treatment routine. We had several patients who were infected with COVID-19 and one related death during treatment in the first few months of the pandemic in São Paulo Brazil.

## INTRODUCTION

Population-based studies suggest that cancer patients undergoing active treatment have a higher propensity for severe events related to coronavirus disease (COVID-19) (1,2). In addition, malignant tumors are more prevalent in older individuals, when comorbidities are usually present. Furthermore, anticancer treatments may result in impairment of the immune system, causing these patients to be even more susceptible to the risks of coronavirus infection (3). In this scenario, the COVID-19 pandemic has presented numerous challenges for cancer care professionals.

Radiation therapy (RT) is one of the cornerstones of cancer treatment. Together with surgery and chemotherapy, it plays an important role in the local control of most malignant tumors and the survival of patients. To maintain an acceptable balance between tumor control and side effects, dose fractionation is the proposed solution. This implies in patients daily visits to the hospital to receive their treatment properly. This practice may present a challenge in the COVID-19 pandemic environment.

A worldwide series of treatment policies and recommendations regarding radiation treatment were published (4-11) and summarized in a review by Vordermark (12). Many of these suggestions considered hypofractionated treatment schedules appropriate to reduce hospital visits, *i.e.*, a reduced number of fractions with a higher dose/fraction that produce the same biological effect as conventional fractionation. Thus, patients may benefit from the same outcomes, with less burden and time consuming. This approach has been gaining momentum in recent years and has raised awareness on the need to minimize patients’ exposure to COVID-19. In addition, safety measures have been exhaustively published during this period and are beyond the scope of this study. The Brazilian Medical Association (*Associação Médica Brasileira* - AMB) has developed guidelines for a comprehensive approach to the COVID-19 pandemic (13).

Hospital das Clínicas da Faculdade de Medicina da Universidade de São Paulo (HCFMUSP) is a public university hospital that comprises a large complex with approximately 2500 beds, and 500 intensive care unit (ICU) beds (including pediatrics and neonatology). The Radiotherapy Department has 10 linear accelerators and one high dose-rate brachytherapy machine for patient treatment. These machines are located in separated buildings (Instituto do Câncer do Estado de São Paulo - ICESP plus a satellite unit) and Instituto de Radiologia - INRAD, respectively). Approximately 400 patients are treated every day. At the end of March 2020, the hospital was urgently remodeled to treat patients who were suspected of or confirmed as having COVID-19.

This study reports the impact of the COVID-19 pandemic on the attendance of patients at the Radiotherapy Department two months after the implementation of specific policies regarding the pandemic.

## MATERIAL AND METHODS

At first, it was established that besides the focus on the hospital treatment of patients with COVID-19, oncological care would remain unchanged. Therefore, a specific contingency plan was developed for radiotherapy at the institution to minimize the displacement of patients during the pandemic. The plan was complementary to the actions and general rules already established at the HCFMUSP Complex. These rules were to be followed by everyone and were to be individualized for the Radiotherapy Services of ICESP and INRAD. The standardization was the same for both Institutes, and specific differences were highlighted. Actions were planned for various perspectives, and the aim was not to impact or compromise the patient’s treatment. Some adaptations were made regarding treatment schedules, follow-up appointments, avoidance of treatment delays and crowded waiting rooms, establishment of priorities for scheduling new cases and treatment, and acceptable delay times for initiating RT. All clinical decisions were discussed at multidisciplinary board meetings. These actions were designed to be modified according to the course of the pandemic.

General recommendations were then established for RT including: 1. cancer treatment should be prioritized over the risk of coronavirus contagion, 2. postponing the initiation of treatment would be acceptable in low-risk, slow-growing disease, and 3. avoidance of irradiation in patients with a short life expectancy (<3-6 months), or in whom tumor response is expected to be low, or in those with a low performance status should be strongly considered (14). Unusual hypofractionated schemes and individualized decisions should be discussed within the team.

Therefore, three priority levels were defined, as follows (Table 1):

I-HIGH: proceed with the RT plan.

II-AVERAGE: consider scheduling RT simulation within three months.

III-LOW: consider scheduling RT simulation after three months.

Besides the well-established treatment protocols followed by the institution, other treatment schedules for the various diagnoses were proposed according to the best evidence available at the time and international recommendations (Table 2).

This study was designed to evaluate the impact of the COVID-19 pandemic on patient attendance at the Radiotherapy Department (ICESP and INRAD). An analysis of the first two-month period (April and May 2020) after the implementation of established policies was carried out, and this period was compared with the same period in 2019.

## RESULTS

**Figure 1:**
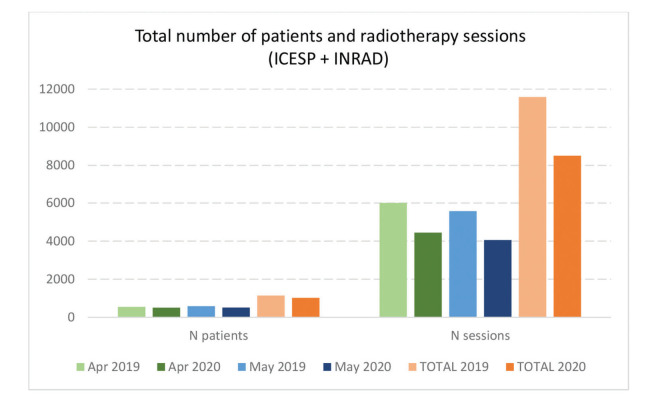
Total number of patients treated and the corresponding number of sessions in April and May 2019, compared to those in April and May 2020.

The attendance numbers in April and May 2019 compared to that in the same period of 2020 are presented in Figures 1 to [Fig f02] 3. Overall, a decrease in the number of treated patients and sessions was observed between the two periods: from 1,145 patients and 11,588 sessions in 2019 to 1,025 patients and 8,506 sessions in 2020. This represented a 10.5% and 26.6% reduction, respectively. Month-wise, there was an 8.6% reduction in the total number of patients in April and a 12.4% decrease in May, corresponding to a reduction in the total number of sessions by 26.0% and 27.2%, respectively (Figure 1).

**Figure 2 f02:**
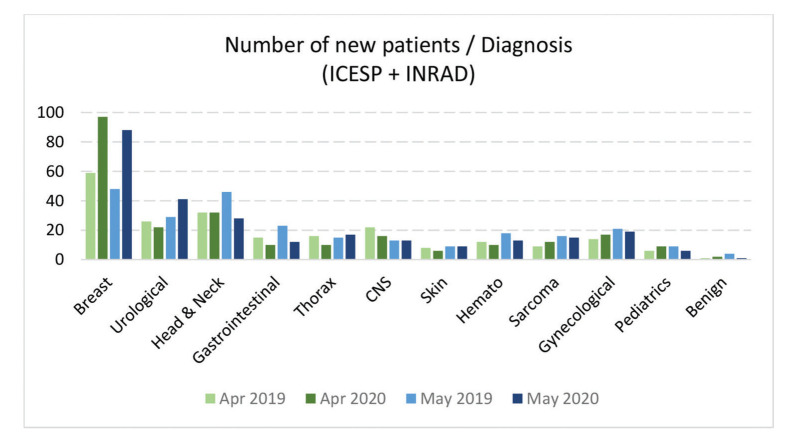
Total number of patients treated in April and May 2019 and 2020, respectively, according to diagnosis. Legend: CNS = Central nervous system tumors.

**Figure 3:**
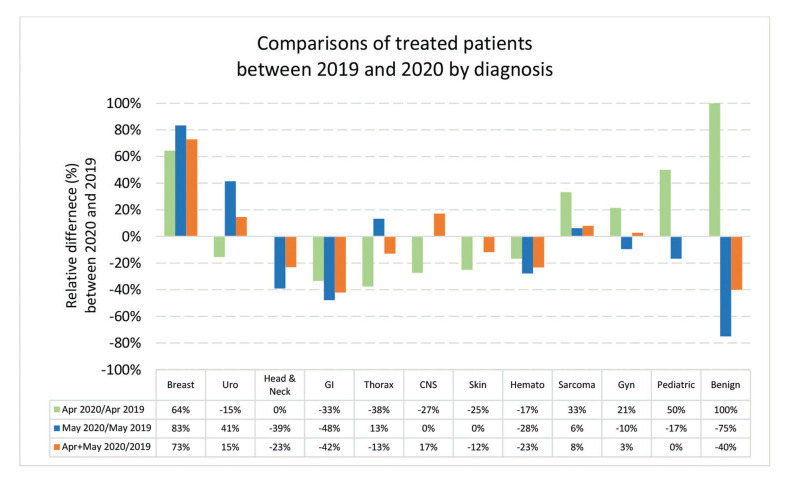
Comparison between April and May 2019 and April and May 2020 by diagnosis of treated patients. Legend: Uro = urological cancers (most prostate); GI = gastrointestinal cancers; CNS = Central nervous system tumors; Hemato = hematological cancers; Gyn = gynecological cancers.

A higher impact was observed in benign diseases and gastrointestinal tumors, followed by head and neck, hematological, thoracic, and skin cancers (reduction ranging from 12% to 23%). In contrast, we observed an increase in the treatment of breast, central nervous system (CNS), urological cancers, sarcomas, and gynecological tumors (ranging from 3% to 73%). Pediatric cancers remained stable (Figures 2 and 3).

Palliative treatments increased by 11.9% in 2020 (from 84 to 94 cases), 2.6% in April, and 20.0% in May, when compared to those in the same period in 2019.

Regarding treatment techniques, external beam irradiation is usually delivered with three-dimensional conformal or intensity-modulated RT (IMRT) for all patients.

**Figure 4:**
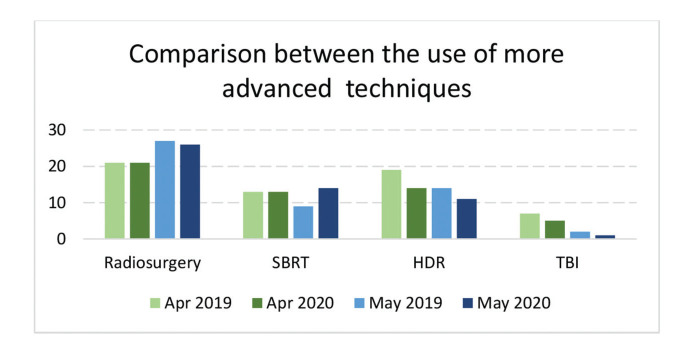
Comparison of the use of other irradiation techniques between the two periods. Legend: SBRT = Stereotactic Body Radiotherapy; HDR = high dose-rate brachytherapy; TBI = Total Body Irradiation.

The variations in the use of brachytherapy or more advanced external beam techniques are presented in Figure 4. A 24.2% decrease was observed in brachytherapy procedures, whereas only a 2.7% decrease was observed for cranial radiosurgery. Stereotactic body radiotherapy (SBRT) increased by 22.7%. Total body irradiation (TBI), indicated for conditioning before bone marrow transplant, showed a 33.3% reduction from 2019 to 2020, 28.6% in April and 50.0% in May.

Eighteen (1.7%) patients were confirmed as having COVID-19 during RT in April and May 2020, three of whom were hospitalized and one of whom died from COVID-19. Of these 18 patients, 12 had their treatment interrupted for at least 15 days from symptom appearance.

Overall, nine staff members were confirmed as having COVID-19, six in April and three in May. Among the 14 medical radiation oncology residents, two were infected, and among the eight medical physics residents, two were infected.

## DISCUSSION

The coronavirus pandemic has led to many changes in public and private health policies worldwide. At our center, a specific challenge was presented as the hospital was designated as a reference center for COVID-19 treatment, and all efforts were directed toward COVID-19 treatment.

Nevertheless, other diseases and morbidities persist in the community, among which is cancer. Cancer is a disease for which diagnosis and treatment cannot be delayed. Therefore, the main policy of the Oncology Department was to continue the treatment of cancer patients. However, specific modifications according to available scientific evidence were permitted. These modifications were implemented to preserve the patient’s outcomes while simultaneously minimizing the risk of COVID-19 contagion in these patients.

Radiotherapy management is particularly complicated in this situation. The cost-effectiveness balance must be evaluated not only regarding cancer treatment but also regarding the risk of infection by patients who need to travel every day to the RT facility for treatment. In addition, during the early stages of the pandemic, the impact of oncological treatment on COVID-19 patients was uncertain.

Safety measures were established to protect patients and staff, and specific recommendations and policies were adopted so as not to impact patient care.

The impact of the pandemic on patient attendance at the RT department was measured during the first two months after the implementation of the proposed RT management protocols. We observed a decrease in the number of treated patients, as expected, of around 10%, with a higher decrease in the number of sessions (26.6%). This reflected a larger number of patients treated with a lower number of fractions, as established in our policies (mean number of sessions/patient decreased from 10.1 in 2019 to 8.3 in 2020). Not analized in this study, however, was the cause for the reduction in the number of patients. It could be due to the fact that a certain number of patients refused treatment initially. A higher rate of absence was also observed because of the fear of the pandemic or the difficulties due to the quarantine laws implemented in the city.

The analysis of the behavior of numbers according to diagnosis revealed a larger negative impact on gastrointestinal tumors treatment, followed by hematological tumors treatments (Figure 3). This may indicate that other treatment options were offered to these patients. There was also the possibility of a delay in irradiation, as observed by the decrease in TBI, for example, that is indicated for hematological disorders. A high decrease in the treatment of benign diseases was expected. However, only a 40% decrease was observed. At our department, most benign diseases are benign CNS tumors that are treated with radiosurgery. Therefore, even though we observed a reduction of almost half among the patients with benign disease, this reduction was not as steep as was expected. This may be because of the possibility of treating these lesions with radiosurgery technique that delivers a single dose or hypofractionated treatments of typically up to five fractions.

Breast cancer represented the largest number of patients and the largest increase in treated patients. For a couple of years, we no longer prescribe the conventional 25 to 28 fractions for these patients. Our current protocol is hypofractionated treatment (15 fractions/3 weeks) for all breast cancer patients, that already allows a lower number of visits to the hospital. In addition, the radiotherapy clinic in our hospital treats only patients referred by the surgery or chemotherapy clinics. However, an exception was made to accommodate the public health system’s requirements for breast and prostate cancer treatments. These patients are referred directly by the system for irradiation at INRAD, even outside of the pandemic scenario. Thus, the attendance of breast cancer patients was not only maintained at the highest level but also showed the highest proportional increase, when related to other diagnoses.

Treatment of urological cancers, mainly prostate cancer, presented a similar behavior, although to a smaller degree, for the same reason (Figure 3).

Treatments with palliative intent presented a proportional increase during the first two months of the pandemic, with more such treatments in May 2020. In absolute numbers, this represented 10 additional patients treated when comparing the two periods. This could be only a circumstantial and/or a seasonal fluctuation. However, the fact that treatment for patients in pain or in emergency situations is always prioritized should not be ignored and may explain or justify this finding.

A limitation of this study is the lack of a detailed analysis of the number of postponed or modified treatments, which was not within the scope of this study. This decision could be held at the referral clinic. In addition, the overall organization of the hospital was completely modified to prioritize COVID-19 treatments. Thus, the impact of this strategy on the number of patients referred to our hospital and to the Cancer Institute (ICESP) was not evaluated.

Regarding the complexity and singularity of this situation in an RT environment and at our hospital, we consider that the decrease in attendance was not substantial and not related to the department’s policies. We consider that it was because of the natural decrease in attendance in the health system overall associated with COVID-19.

However, the pandemic growth is stabilizing in the state of São Paulo (16), and HCFMUSP is slowly discontinuing the “COVID-19 strategy.” Therefore, we expect that the decrease in the number of treated patients observed during April and May 2020 will gradually normalize to the baseline levels. A positive finding was that among the 1,025 patients treated during April and May 2020, only 18 patients (1.7%) were infected with COVID-19.

## CONCLUSION

The COVID-19 pandemic presented numerous impacts on health systems, social life, and human behavior worldwide. It remains a challenge for local communities. A decrease in the number of treated patients was observed in our radiotherapy department despite our efforts to keep the treatment routine as normal as possible. Nevertheless, a higher decrease in the total number of sessions was detected, indicating a smaller number of fractions/patients overall. We had relatively few patients who were diagnosed as having COVID-19, and there was one related death during treatment in the first few months of the pandemic in São Paulo, Brazil.

## AUTHOR CONTRIBUTIONS

Carvalho HA contributed in conception, design, analysis and drafting the article. Vasconcelos KGMC and Gomes HC contributed in acquisition, interpretation of data and final approval. Salvajoli V contributed in drafting the article and final approval.

## Figures and Tables

**Table 1:** Priority levels for treatment indication and RT delivery during the COVID-19 pandemic, according to tumor diagnosis.

Priority levels		
I - HIGH Proceed with RT plan	II - AVERAGE Consider scheduling RT simulation within 3 months	III - LOW Consider scheduling RT simulation after 3 months
1) Urgencies 2) Cases where RT is the main treatment: • NSCLC • Uterine cervix • Head and neck • Anal canal 3) Pediatric tumors 4) Brain metastases 5) High-risk lymphomas 6) Glioblastomas 7) Rectal cancer 8) Selected palliative treatments	1) Breast 2) Endometrium 3) Gastrointestinal	1) CNS low grade 2) Prostate (low or intermediate risk in hormone therapy, and high risk with only one risk factor) 3) Benign tumors 4) Elderly with favorable tumors* • Low risk breast • Skin • Prostate 5) Multiple brain metastases* *consider NOT treating

Legend: NSCLC = non-small cell lung cancer; CNS = central nervous system.

**Table 2:** Summary of the proposed changes in radiotherapy management for various tumors.

Cancer site	RT schedule	Postpone RT start	Interrupt RT*
NSCLC	SBRT: no changes Consider hypofractionation for stage III without concomitant chemotherapy Adjuvant treatment not recommended	No Consider postponing SBRT in indolent tumors	Yes
SCLC	Limited disease: no changes Extensive disease: may consider PCI and thorax consolidation if response	No	Yes
Breast	Keep 15 fractions Consider 5 fractions (26 Gy/5 fractions) for selected patients (>60 years, breast only RT) Consider omission of RT for low risk elderly (>70 years) patients	Yes, up to 16 weeks	Yes
Uterine cervix	No changes	No	No
Endometrium	Stage I, G2-G3 intermediate risk or Stage II, and stage III: consider no RT according to comorbidities	Yes	Yes
Vulva & Vagina	No changes	No	Yes, if RT adjuvant
Head & Neck	No changes	No	No
CNS	No changes Hypofractionation for glioblastoma (research protocol)	Yes, low grade	
Prostate	No changes Favor hypofractionation	Yes	Yes
Bladder & Testis	No changes Consider hypofractionation for bladder	No	No
Esophagus	No changes	Yes, up to 3 months in indolent disease	Individualize
Stomach	No neoadjuvant or adjuvant RT	Yes, up to 3 months	Individualize
Pancreas	Consider neoadjuvant SBRT (research protocol) No neoadjuvant or adjuvant RT (Neoadjuvant CT maintained)	No	Individualize
Rectum	No changes or consider neoadjuvant RT in 5 x 5 Gy (ECOG 0-2) followed by CT in the interval between RT and surgery	No	Individualize
Anal canal	No changes	No	Individualize
Pediatrics	No changes	No	Individualize
Cranial Radiosurgery	No changes	Yes, only benign diseases	No
SBRT not for lung cancer	No changes	No	No

* Radiotherapy interruption should be considered if the patient is a suspected or confirmed case of COVID-19 or according to clinical conditions if the treatment is to proceed. If interruption was indicated, the recommendation was for 15 days of interruption and then to restart with a dose compensation if applicable. The impact of treatment gaps and dose compensation should be evaluated according to Gay et al. 2019 (15).

Legend: RT = radiotherapy; NSCLC = non-small cell lung cancer; SCLC = small cell lung cancer; CNS= central nervous system; SBRT = stereotactic body radiotherapy; RT = radiotherapy; CT = chemotherapy; ECOG = Eastern Cooperative Oncology Group performance status.
